# Should a Doctor Tell?

**Published:** 1920-07-03

**Authors:** 


### THE PUBLIC WELL-BEING.

### Should a Doctor Tell?

### Some Considerations of his Delicate' Position.

"*Every righteous physician regards his practice as a social service, a means not only of bodily but also of social reconstruction, and of moral and intellectual health. Thus medicine will become more and more a binding force in societies, a bond between nations, and a purification of the world.*"—(Sir T. Clifford Allbutt, in his presidential address to the British Medical Association.)

The difficult question, so often debated, of the relation which ought to exist between a medical man and his patients in regard to secrecy was a subject of debate at the meeting of the British Medical Association at Cambridge. It was considered with special regard to venereal diseases, and the Association reiterated its opinion that the "medical practitioner should not, without his patient's consent, voluntarily disclose information which he has obtained from such patient in the exercise of his professional duties." This is generally accepted by the lay press as a sound decision, though there have been some criticisms of its omnibus character. There was, in fact, some criticism in that respect during the Association's discussion. It is perhaps best indicated in the question by Dr. C. Sanders, Stratford, London, who asked: "Does that resolution mean this, that we are, as a profession, to allow a bounder to live and his wife and child to die?" Dr. Bishop Harman, of London, put the same point in another way. If a man were affected by venereal disease, he said, and the doctor held his peace, he would be affecting somebody else. In that case, if he could save persons from death or a life of misery and did not do so, and the circumstances became known, would he not be liable to have a case brought against him by the person injured? Anyone taking action in such a case would, in Dr. Harman's opinion, win hands down. His view was that they could not say that in no circumstances would they disclose information. The discussion seemed rather to tend towards this idea, that while the general rule laid down should be observed, it must necessarily be elastic and subject to the pressure of particular circumstances. The medical profession has its general guidance, and doubtless any member of it could get authoritative advice in special cases.

#### The Marriage Question.

The few points just mentioned are sufficient to indicate how delicate a position the doctor occupies. Broadly speaking, it is obvious that patients are entitled to demand that a doctor should respect their confidence, and one of the endeavours in the campaign against venereal disease is to make treatment such that a patient will not hold back for fear of his shame being disclosed to the little world in which he lives and moves. Secrecy can be the only guarantee of success in such a campaign as this, and it is quite certain that if the impression were to get abroad that a patient's confidence was only to be respected with considerable mental reservations, the victims of venereal disease would shrink from approaching members of the medical profession in this matter, and patients generally would discuss their condition with their doctor without candour and so conceal many circumstances essential to correct diagnosis or proper treatment. It is a common saying, and a true one, that a doctor stands in the relation of a father confessor to his patient; and that fact, which proves how high and unquestioned is the tradition that the British doctor, specialist and general practitioner alike, preserves inviolate the personal revelations committed to his keeping, is a by no means unimportant element in the maintenance of public health. Let that trust once be impaired and the relationship of doctor and patient will be changed vastly for the worse.

On the other hand, every doctor knows that the occasion frequently arises when concealment is bad for the patient or for those immediately connected with him. The position is often specially difficult in the case of a young man or a young woman about to be married. Leading out of the discussion on this point, it has been suggested by more than one eminent member of the profession within the last few days that the problem would be solved by a law making it compulsory upon every man about to be married—why not every woman also?—to obtain a certificate of health, not necessarily certifying him to be in an A1 category, but declaring him to be free from organic or communicable disease. This would certainly relieve the doctor of an anxious responsibility, and at the same time do a great deal towards creating the conditions requisite for producing a vigorous race; but we confess our doubt as to whether public opinion in the country would at present accept a condition that would probably be regarded as in the last degree irksome and as impinging offensively on individual liberty. For the time being, it is to be supposed, the doctor must continue to depend upon his own common sense and discretion. In those comparatively rare instances in which he is brought up sharply against some gross crime against society he will have no trouble in following the dictates of his conscience, but in the far more numerous cases where a delicate point of honour is involved he will have to rely upon his own judgment in deciding to uphold, or depart from, the safe and almost invariable rule that a doctor must not tell.

#### Telling the Patient.

One aspect of the question which has an even more intimate bearing in the everyday life of the doctor was not touched upon in the debates of the Medical Association. That is, the perennial problem how far it is legitimate or wise for the patient

**THE PUBLIC WELL-BEING**—(*continued*)

himself to be told his true condition, if it is a bad one. We have in mind the case of a young man of high-strung temperament and brilliant capacity who a few years ago was discovered by a specialist to be suffering from disseminated sclerosis. The specialist withheld the fact from his patient, but informed the relatives, and strongly advised them to maintain silence. Unfortunately a couple of years later, the young man found out by accident the nature of the incurable disease which had attacked him, and the shock of this chance discovery was disastrous to his peace of mind and his general condition. Should his condition have been tactfully revealed to him in the first place, or in spite of the accident was concealment the right course? It is impossible to say without full knowledge of all the circumstances. Here, again, the doctor must employ common sense and discretion, with the additional factor of a knowledge of psychology. The psychosis of a case cannot be disclosed unless the personality of the patient and his personal history are known clearly to the doctor. The vital facts cannot be known to him unless he has the complete confidence of the patient. He cannot secure the complete confidence of his patient unless the traditions of secrecy remains a *sine qua non* in all the dealings of the medical profession with the members of the public who go to them for the cure of their bodies. The argument, therefore, seems to lead inevitably to the general conclusion on the main question that in the interest alike of the profession and the public, the medical practitioner should not without his patient's consent, save in circumstances of a wholly exceptional character, "voluntarily disclose information which he has obtained from such patient in the exercise of his professional duties."

